# Development of Methods for the Selective Measurement of the Single Amino Acid Exchange Variant Coagulation Factor IX Padua

**DOI:** 10.1016/j.omtm.2018.05.004

**Published:** 2018-06-28

**Authors:** Alfred Weber, Andrea Engelmaier, Dirk Voelkel, Robert Pachlinger, Friedrich Scheiflinger, Paul E. Monahan, Hanspeter Rottensteiner

**Affiliations:** 1 Shire, Process Development & Technical Services, Benatzkygasse 2-6, Vienna, Austria; 2 Shire, Global Research, Donau-City-Str. 7, Vienna, Austria; 3 Shire, 650 East Kendall Street, Cambridge, MA, USA

**Keywords:** bioanalytical methods, hemophilia, gene therapy, clinical samples, enzymes, biomedical analysis

## Abstract

The description of hyper-functional factor IX (FIX) Padua triggered the development of BAX 335, an AAV8-based hemophilia B gene therapy vector designed to compensate for low FIX protein expression levels by expressing the FIX Padua variant, thereby reducing the exposure to viral vector. The presence of inactive FIX protein at baseline hindered conventional FIX:Ag ELISA from contributing to a more profound understanding of clinical data from the BAX 335 Phase 1/2 study (ClinicalTrials.gov: NCT01687608). By applying phage display technology, a Fab2 mini-antibody selectively binding to FIX Padua was developed and used to establish a FIX Padua-specific ELISA. The assay adequately performed, utilizing human and monkey plasma samples, and enabled the selective quantification of FIX Padua protein in human plasma samples from the BAX 335 trial. The mini-antibody also allowed the development of a chromogenic FIX Padua-specific activity assay, which adequately performed in human and mouse plasma. Collectively, the isolated FIX Padua-specific mini-antibody enabled the development of transgene-product-specific assays, which should improve the monitoring of hemophilia B gene therapies. The approach applied here for FIX Padua could be leveraged to develop variant-specific activity assays for other therapies where highly active enzymes are instrumental in achieving therapeutic levels of the transgene product.

## Graphical Abstract

**Figure:**
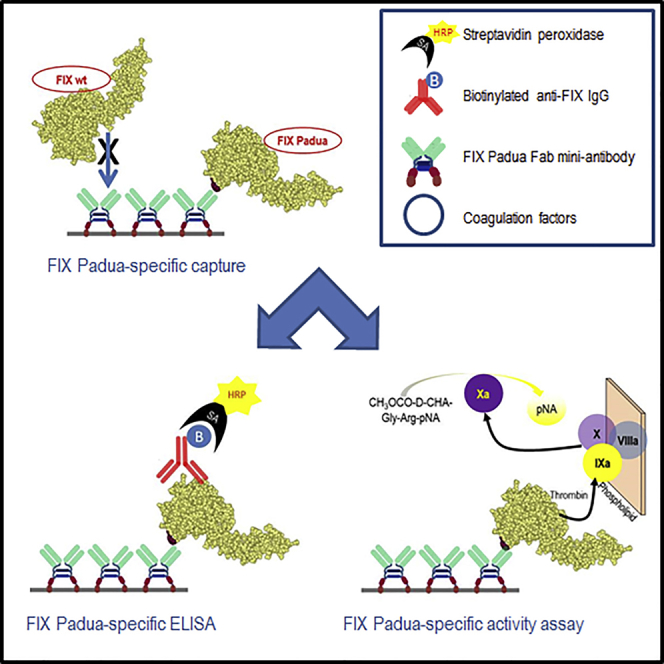

## Introduction

Severe to moderate hemophilia B, an X-linked hereditary deficiency of functionally active human coagulation factor IX (FIX), is characterized by spontaneous or traumatic bleeding episodes.^1^ If not treated adequately by FIX replacement therapy, hemophilia B can result in disability and death. Efficient replacement therapy is based on the administration of plasma-derived or recombinant FIX (rFIX) preparations. Current established prophylaxis regimens comprise intravenous infusions 2–3 times weekly, the frequency being determined by the half-life of the exogenous FIX product (approximately 18–24 hr). To overcome issues related to the required frequent dosing, efforts were taken to develop modified FIX preparations with longer half-lives.^2^, ^3^ Thus, three differently modified FIX preparations with prolonged circulatory half-lives have been made available recently for hemophilia B treatment: a recombinant rFIX-immunoglobulin G1 (IgG1) Fc fusion protein, a glycoPEGylated rFIX preparation, and an rFIX preparation linked to human serum albumin.^4^, ^5^, ^6^ Despite this remarkable progress in treatment modalities, all modified rFIX preparations have still to be administered persistently, albeit at a clearly lower frequency to obtain protective circulatory FIX levels (>1%). Thus, attempts focused on gene therapy to cure hemophilia B are still warranted.

Early data published by Kay et al. in 1993 showed sustained partial correction of FIX deficiency in FIX-deficient dogs using a retroviral vector, and since then, ongoing development of this approach took place.^7^, ^8^, ^9^, ^10^, ^11^ The study published by Nathwani et al. represented a milestone providing evidence that using an adenovirus-associated virus (AAV) vector-mediated transfer of the FIX gene resulted in the sustained expression of FIX in hemophilia B patients.^12^ This basic concept was enriched by using the naturally occurring single amino acid exchange variant FIX Padua, where leucine is substituted for arginine at position 338 (FIX Padua, R338L) as the expression target.^13^ This gain-of-function mutation shows specific activity up to 10-fold higher than that of the FIX wild-type.^13^ Nonclinical studies, one of which supports an ongoing clinical trial, supported the hypothesis that expression of this FIX variant with higher specific activity will result in adequate FIX activities, despite low protein expression levels.^14^, ^15^, ^16^ Current achievements in this experimental field of hemophilia B treatment are reviewed.^17^, ^18^, ^19^

Expression of the FIX Padua variant has been studied in the BAX 335 phase 1/2 gene therapy trial in adults with hemophilia B (AskBio009, NCT01687608), using an AAV8-based gene transfer vector designed to enable liver-specific expression of a FIX Padua cDNA. Assessment of the gene therapy’s success would benefit from analytical methods that enable specific detection of the vector-derived transgene product, particularly in the background of functionally inactive FIX, as present in cross-reactive-material-positive (CRM^+^) hemophilia B patients, who are counting for up to 30%–40%.^20^ Therefore, we developed a Fab2 mini-antibody that selectively recognizes the FIX Padua protein harboring a single amino acid exchange and used this antibody to set up FIX Padua-specific methods.

## Results

### Screening for a FIX Padua-Specific Antibody

The initial screening with a linear peptide and a structural peptide containing the FIX Padua-specific sequence identified 94 binders, which, after sequencing, turned out to represent 34 unique antibody candidates. When these candidates were expressed, purified, and retested, six FIX Padua-binding candidates remained. From the four candidates that emerged from the structural peptide approach, two were cross-reactive to wild-type FIX, and the other two failed to detect FIX Padua in a 20% human plasma matrix. Also excluded was one of the two candidates isolated from the linear peptide approach, as it was cross-reactive to human factor II (FII). Only candidate Ab42 bound highly specifically to FIX Padua, and its determined affinity of K_D_ (dissociation constant) of 3.4 × 10^−9^ M^−1^ was similar to that of a typical polyclonal anti-FIX antibody (K_D_ = 3.1 × 10^−9^ M^−1^). It also showed no reactivity to wild-type FIX or other human plasma proteins, including FII and factor X (FX). Ab42 was, therefore, expressed as Fab2 mini-antibody and selected for further method development.

### Setting Up of a FIX Padua-Specific ELISA

**Figure 1:**
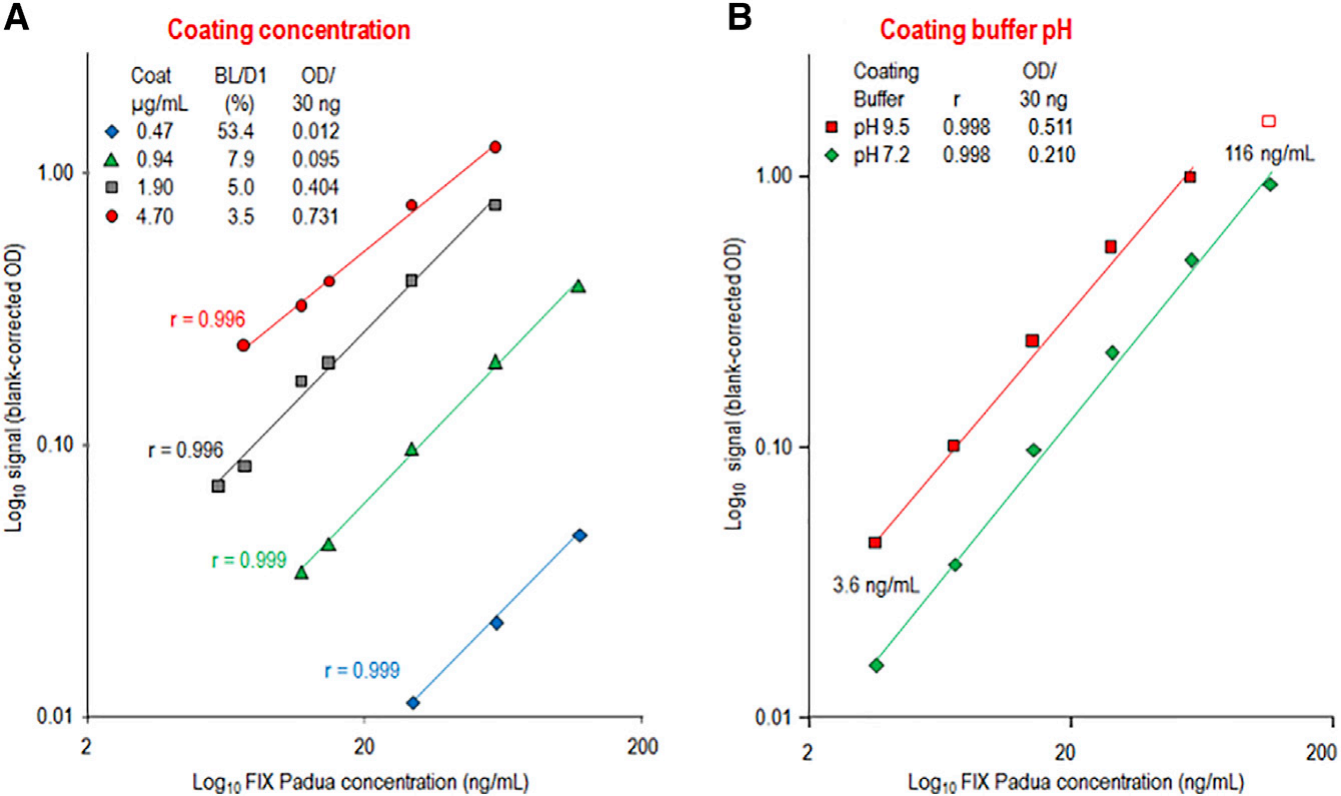
Coating Conditions for the FIX Padua-Specific Fab2 Mini-antibody (A) The concentration-response curves obtained for the different coating concentrations. (B) Comparison of the curves obtained with PBS (pH 7.2) and carbonate (pH 9.5) used as coating buffer.

The use of a Fab2 mini-antibody instead of a full-length polyclonal or monoclonal antibody called for a check of the coating conditions. The coating concentration showed a clear influence on the signals obtained for an rFIX Padua preparation, as the signal height proportionally increased with the coating concentration (Figure 1A). In particular, the assay sensitivity expressed as the quotient of the blank-corrected optical density (OD) per 30 ng rFIX Padua increased by more than 50 times from 0.012 to 0.731, with the coating concentration increasing from 0.47 to 4.7 μg/mL. Therefore, to obtain sufficient assay sensitivity, the coating concentration of the Fab2 mini-antibody should be at least 0.9 μg/mL; this concentration was used for the final ELISA setup, as it resulted in an appropriate range of adequate signal to concentration relation.

The influence of the coating buffer (CB)’s pH on the sensitivity of the assay was checked by comparing a neutral-pH PBS buffer (pH 7.2) with an alkaline-pH carbonate buffer (pH 9.5) at a fixed coating concentration of 1.9 μg/mL. The attributes describing the quality of the curve fitting were similar for both coating conditions, but coating at a pH of 9.5 resulted in a sensitivity that was increased more than two times (Figure 1B) and was, therefore, selected for the final method.

**Figure 2:**
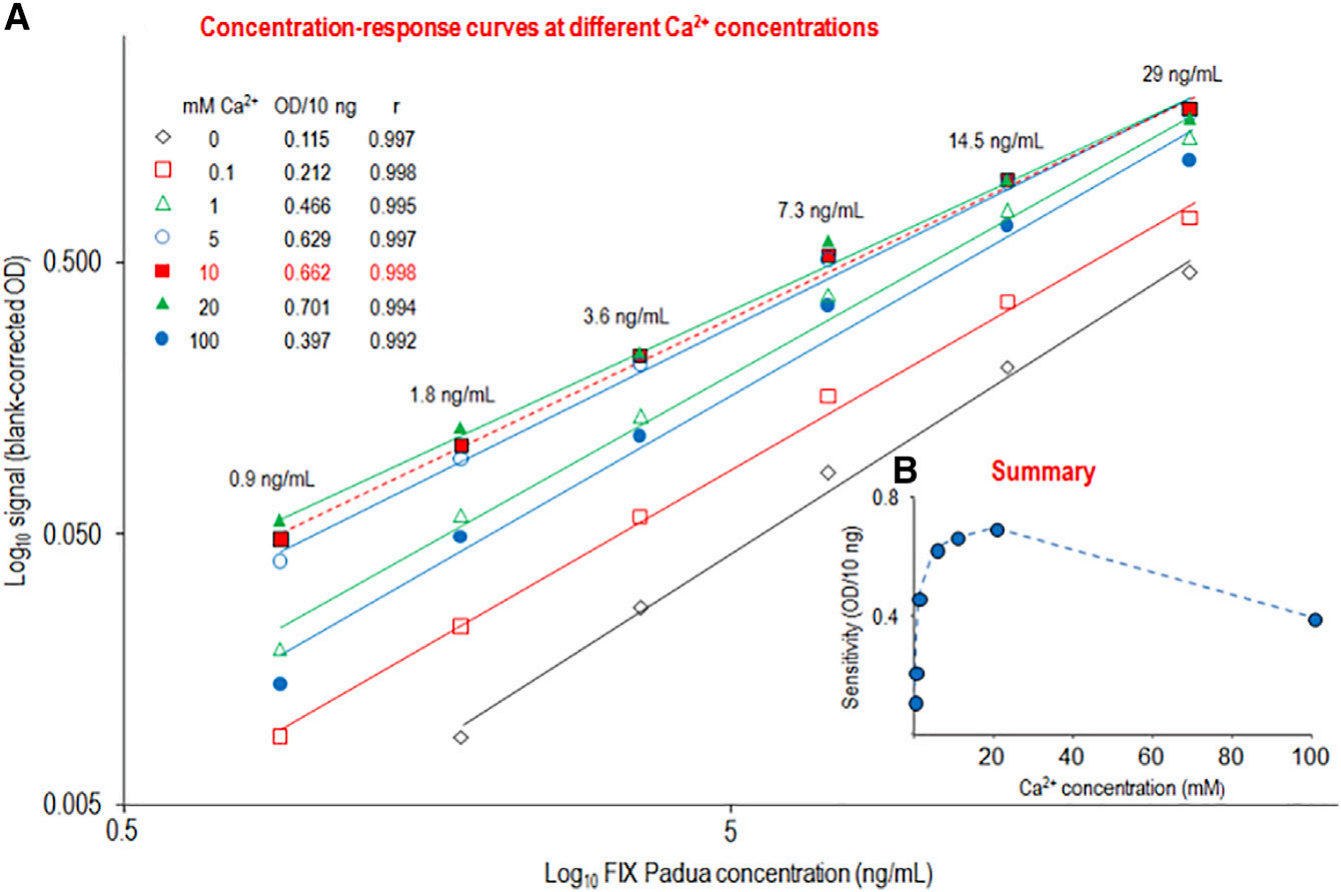
Influence of Ca^2+^ on Assay Sensitivity (A) The dose-response curves obtained at different Ca^2+^ concentrations. (B) A graphical summary on the influence of Ca^2+^ plotting the sensitivity parameter OD/10 ng against the Ca^2+^ concentration of the dilution buffer.

Initial data suggesting Ca^2+^ to profoundly influence the assay (data not shown) prompted us to explore the effect of Ca^2+^ at concentrations ranging from 0 to 100 mM (Figure 2). Compared to a setup lacking calcium, the assay sensitivity increased 4-fold already at a Ca^2+^ concentration of 1 mM and peaked at 20 mM, whereas a further increase to 100 mM caused the sensitivity to decrease again (Figure 2A). Figure 2B further shows that the incremental sensitivity increase flattened at higher Ca^2+^ concentrations. Therefore, it was decided to include 10 mM Ca^2+^ in the dilution buffer (DB).

Therefore, the final DB, which was also used as blocking buffer, contained 100 mM HEPES, 100 mM NaCl (pH 7.2), 10 mM CaCl_2_, 10 mM benzamidine, 0.05% Tween 20, and 0.5% biotin-free BSA. Benzamidine was included in the DB as a nonspecific protease inhibitor to protect against proteolytic degradation. Biotin-free BSA (0.5%) was included as inert protein to prevent protein adsorption to surfaces, which was considered particularly relevant, given the expected assay sensitivity. Bound FIX Padua was detected by the sequential addition of a biotinylated detection antibody and streptavidin peroxidase, followed by the quantification of the chromogenic activity of the peroxidase at 450 nm.

### Calibration Curves of the FIX Padua ELISA

**Figure 3:**
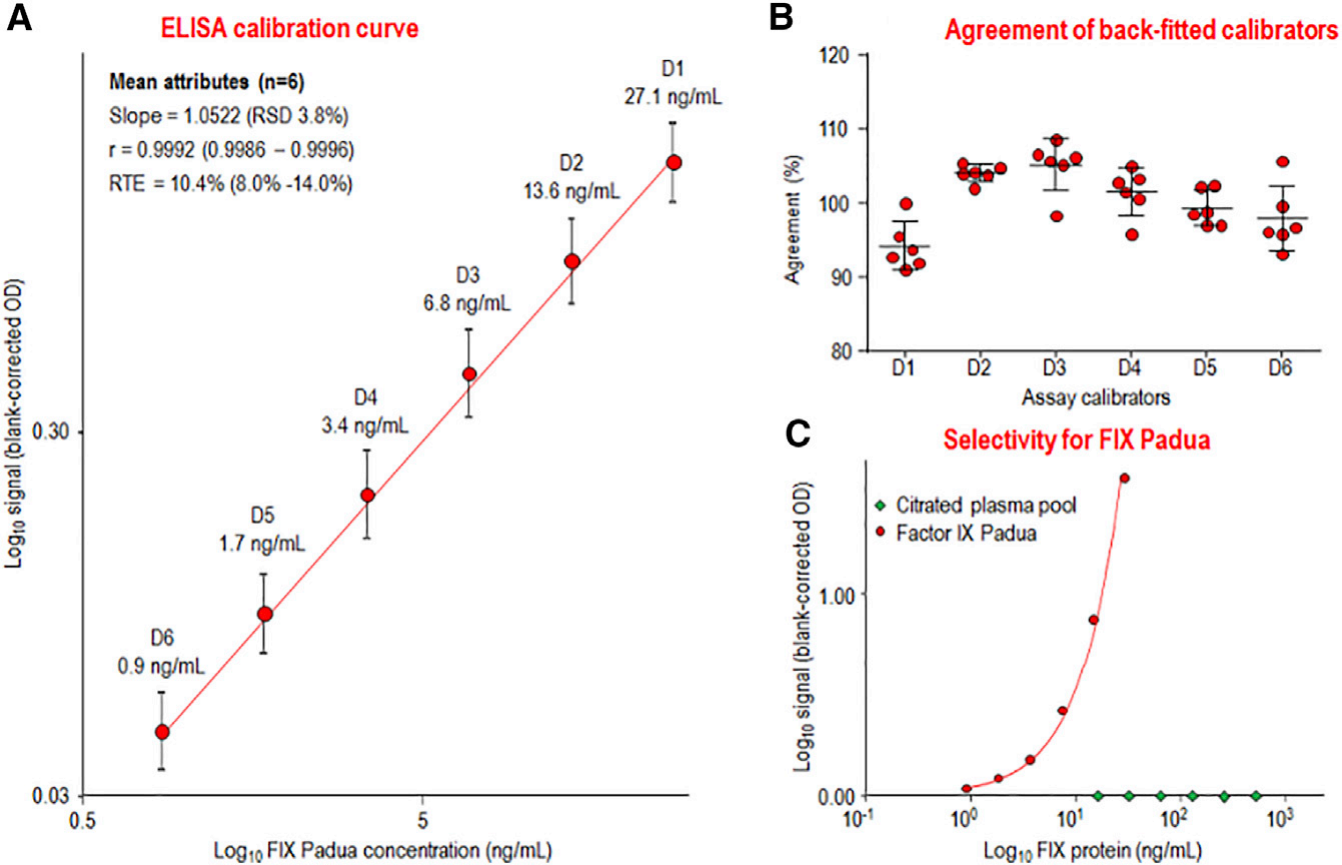
FIX Padua ELISA Calibration Curve and Specificity Check of the ELISA (A) The mean (n = 6) calibration curve of the FIX Padua ELISA. Error bars indicate the single SD of the blank-corrected ODs. (B) The agreement of the back-fitted assay calibrators with their nominal concentrations. (C) Comparison of the concentration-response curves for FIX Padua and normal human plasma.

The calibration curve ranged from 0.9 to 27.1 ng/mL rFIX Padua (Figure 3A). The curves were reproducible and accurate, as shown by the low relative SD (RSD) determined for the mean slope and the low mean relative total error (RTE), ranging from 0.9986 to 0.9996 and from 8.0% to 14.0%, respectively. These data confirmed the suitability of the log-log calibration curve model. In addition, the agreement of the back-fitted assay calibrators with their nominal concentrations (Figure 3B) met the 80% to 120% range defined for ligand-binding assays by the European Medicines Agency (EMA) guideline for bioanalytical method validation.^21^ Figure 3C also shows the results of the selectivity check; in particular, the dilution-response curves for rFIX Padua and human plasma. These data clearly confirmed the selectivity for FIX Padua: normal human FIX, present at a concentration of 1.13 IU/mL (5 μg/mL),^22^ did not show any response, even when present at an excess more than 500-fold over FIX Padua.

### Parallelism Study in Human Plasma and Assay Performance in Cynomolgus Plasma

**Figure 4:**
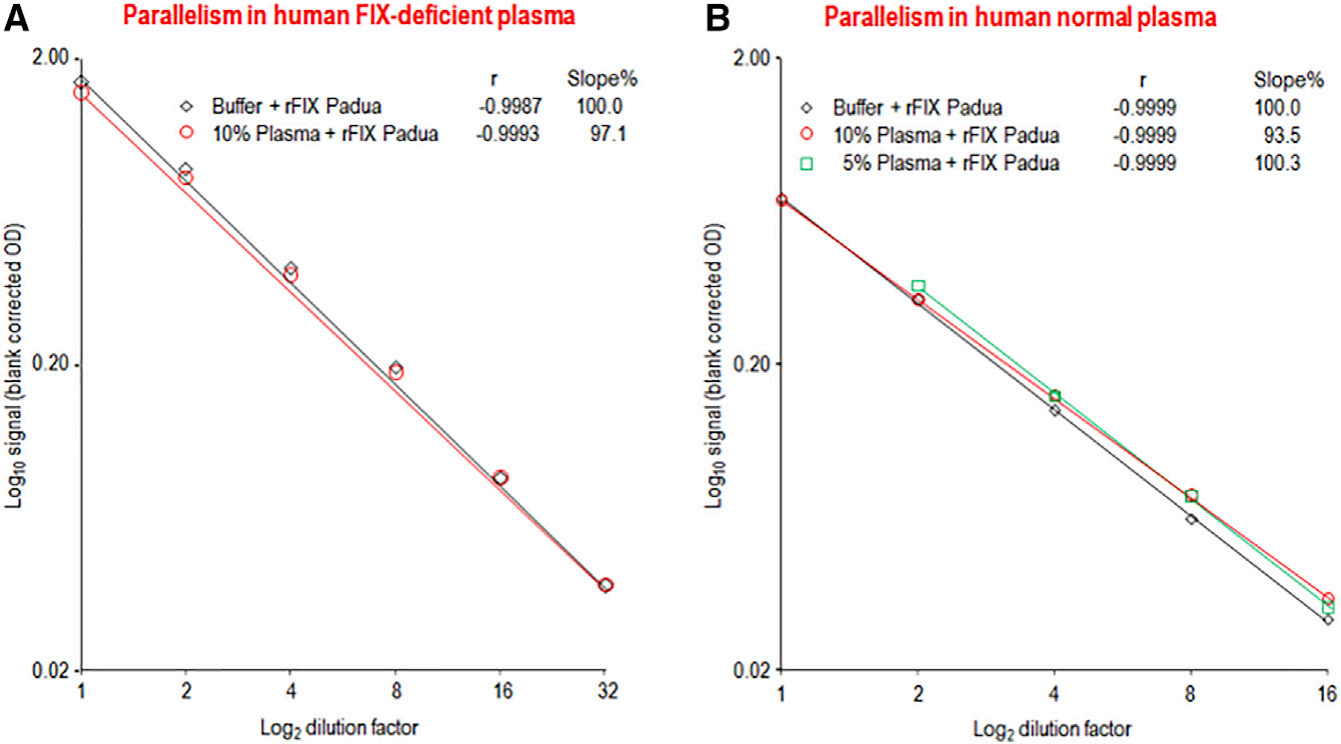
Parallelism Study for the FIX Padua ELISA (A and B) The dilution-response curves are shown for human FIX-deficient plasma (A) and normal human plasma (B).

The parallelism study was carried out not only in human FIX-deficient plasma but also in human normal plasma, mimicking the presence of ELISA-reactive FIX protein, as this will be the case for CRM^+^ hemophilia B patients. The data demonstrated that the 10% plasma matrix had no influence on the dilution-response curve obtained for rFIX Padua, which was parallel to that obtained in buffer with the marginal difference in slope of 2.9% (Figure 4A). Together with the good correlation coefficient determined, these data reasonably justified the preparation of the calibration curve in buffer. Furthermore, the minimum dilution of 1/10 for the analysis of human citrated FIX-deficient plasma was confirmed. The mean recovery of spiked rFIX Padua (230 ng/mL; n = 3) was 113.6%. A similar dataset was generated for citrated human normal plasma, diluted to 10% and 5%. The correlation coefficients were high for all dilution-response curves (Figure 4B). The slopes of the dilution-response curves obtained for the plasma matrix samples differed by less than 7% from that obtained for the dilution-response curve obtained in buffer. The recoveries of spiked rFIX Padua were 112.3% and 116.6% in the 10% and 5% diluted human reference plasma matrix samples, respectively. Initial intra-run precision data, obtained for rFIX Padua in buffer (n = 3) and in FIX-deficient plasma (n = 4), were acceptable with RSDs of 0.7% and 1.8%, respectively. All values met commonly accepted bioanalytical acceptance criteria for this accuracy parameter.^21^

**Figure 5:**
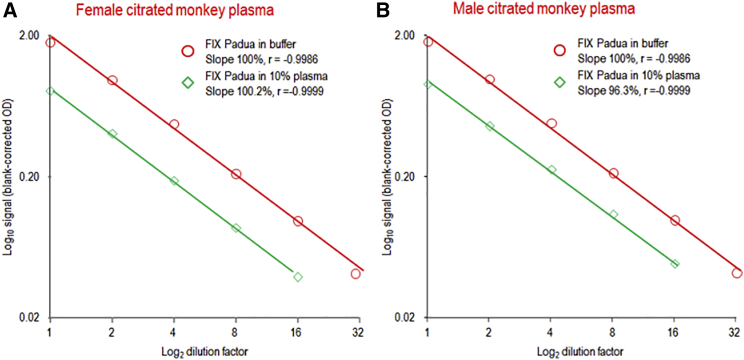
Parallelism Study for the FIX Padua ELISA in Citrated Cynomolgus Monkey Plasma (A and B) The dilution-response curves are shown for female (A) and male (B) normal citrated cynomolgus plasma samples.

Cynomolgus monkey FIX shows a high degree of homology to human FIX so that conventional ELISAs usually cannot discriminate between human and monkey FIX. It was, therefore, of interest to see whether a specific FIX Padua measurement can be achieved in this nonclinical test model. Two citrated cynomolgus monkey plasma samples were measured without and after spiking with rFIX Padua. The FIX Padua levels were <8.5 ng/mL in the plasma samples, using a minimum dilution of 1/10. However, the recovery of spiked rFIX Padua was 91.3% and 105.3% in the female and male plasma samples, respectively. The dose-response curves obtained for the spiked samples were highly linear and parallel to that of the assay standard with their slopes, differing by less than 5% from that of the calibration curve. Thus, the method proved feasible also in the matrix of citrated cynomolgus monkey plasma (Figure 5).

### Performance of the FIX Padua ELISA Using Human Plasma Samples of the BAX 335 Clinical Trial

The performance of the FIX Padua ELISA was further validated by assaying citrated plasma from screening samples of patients during the conduct of the phase 1/2 open-label clinical trial of BAX 335 (ClinicalTrials.gov: NCT01687608). This confirmation included spike recovery with rFIX Padua and parallelism evaluation of the dilution-response curves obtained for these spiked citrated plasma samples and the assay standard. Table 1 shows the recovery data: the mean recovery of spiked rFIX Padua (27.2 ng/mL) was 92.4%, with individual recoveries ranging from 86.7% to 100.7%. Two of these patients’ plasma samples were further used to show linearity on dilution. Their dilution-response curves were parallel to that of the assay standard. In particular, their slopes were 101.7% and 108.9% of that of the assay standard curve. These data confirmed the accuracy, specificity, and parallelism of the FIX Padua ELISA and warranted its use for the detection of FIX Padua transgene product in plasma samples from patients treated with BAX 335.

**Table 1:** Spike-Recovery Data for the FIX Padua-Specific ELISA

Sample No.	Neat FIX Padua Concentration (ng/mL)	Concentration after Spiking (ng/mL)	% Recovery
1	<8.5	25.9	95.5
2	<8.5	24.5	90.3
3	<8.5	23.9	88.1
4	<8.5	24.0	88.5
5	<8.5	25.4	93.7
6	<8.5	23.5	86.7
7	<8.5	26.0	95.9
8	<8.5	27.3	100.7

The FIX Padua concentration spiked to patients’ citrated plasma samples was 27.1 ng/mL. The samples were measured in the final dilution of 1/10. Recovery was calculated according to (found concentration/nominal concentration) × 100.

A representative selection of 29 citrated plasma samples from two CRM^+^ and two CRM^−^ patients drawn at different time points post-dosing was, therefore, analyzed with the FIX Padua-specific ELISA described here and with standard methods for measuring FIX coagulation activity and FIX antigen. The results for the two standard assays were obtained in international units per milliliter, and those for the FIX Padua ELISA were obtained in nanograms per milliliter. To enable for appropriate data comparison, the results of the FIX Padua ELISA, obtained in nanograms of FIX Padua per milliliter, were transformed to activity units, based on the specific activity of the rFIX Padua preparation used as assay calibrator (2,310 IU FIX per milligram). Thus, 1 ng rFIX Padua equals 2.31 mIU clotting activity. This reasonable assumption does not take into account possible differences in specific activity between the Chinese hamster ovary (CHO)-cell expressed FIX Padua standard and the transgene product.

The FIX Padua concentrations measured with the FIX Padua-specific ELISA matched well with the FIX activity concentrations (Table 2); a linear correlation was seen for the two parameters (y = 0.7587–0.0022; R^2^ = 0.97).

**Table 2:** Test for Correlation of FIX Padua-Specific ELISA Data with Standard FIX Parameters in a Collection of 30 Representative Clinical Samples from the BAX 335 Trial

Sample No.	CRM	FIX Padua		FIX Activity (IU/mL)	FIX Antigen (IU/mL)^b^
		ng/mL	U/mL^a^		
1	+	53.3	0.123	0.091	0.883
2	−	27.3	0.063	0.053	0.016
3	−	184	0.425	0.320	0.036
4	+	99.1	0.229	0.197	0.907
5	−	<8.5	<0.020	0.016	<0.009
6	−	27.9	0.064	0.054	0.939
7	−	92.0	0.213	0.154	0.040
8	+	91.5	0.211	0.163	0.817
9	+	127	0.293	0.259	1.000
10	+	332	0.767	0.585	0.881
11	+	171	0.395	0.266	0.780
12	+	155	0.358	0.252	0.851
13	+	118	0.273	0.138	0.864
14	+	91.3	0.211	0.162	0.867
15	+	56.7	0.131	0.091	0.892
16	−	76.5	0.177	0.108	0.020
17	−	115	0.266	0.205	0.030
18	−	134	0.310	0.233	0.030
19	−	22.2	0.051	0.047	0.012
20	+	64.9	0.150	0.108	0.888
21	+	48.2	0.111	0.085	0.925
22	+	103	0.238	0.193	0.945
23	+	115	0.266	0.199	0.843
24	−	<8.5	<0.020	0.010	<0.009
25	+	119	0.275	0.234	0.900
26	+	118	0.273	0.204	0.846
27	−	<8.5	<0.020	0.008	<0.009
28	+	152	0.351	0.234	0.895
29	+	234	0.541	0.440	0.837
30	+	<8.5	<0.020	0.009	0.952
31	−	<8.5	<0.020	0.011	0.022

Collection included samples from 4 patients (2 CRM^+^, 2 CRM^−^) from the BAX 335 trial (NCT01687608) at time points post-dosing ranging from 7 to 279 days. Samples 30 and 31 were taken before dosing. The results of the FIX Padua ELISA and the FIX activity assay had an R^2^ of 0.97.

a FIX Padua data were transformed to FIX activity in units per milliliter: 1 ng/mL FIX Padua Ag equals 2.31 mU/mL clotting activity.

b FIX antigen data are shown in international units per milliliter; 1 IU resembles the amount of FIX present in 1 mL normal plasma (5 μg/mL).

Furthermore, the presence of FIX cross-reactive material had no influence on the specific FIX Padua measurement but strongly biased the conventional FIX protein measurement toward detection of functionally inactive FIX. The FIX Padua ELISA data, therefore, provided unambiguous evidence that expression of FIX Padua was elicited in the patients treated with BAX 335, and they support implementation of the assay in FIX Padua-based hemophilia B gene therapy trials.

### FIX Padua-Specific Chromogenic Activity Assay

The availability of a FIX Padua-specific Fab2 mini-antibody triggered the idea of also developing a specific activity assay for FIX Padua. Such an assay, although not mandatory for clinical analysis, would nevertheless provide valuable additional information for animal systems with an intact FIX locus (i.e., with measurable FIX activity). Currently established FIX activity assays cannot discriminate between endogenous FIX and FIX Padua expressed after gene transfer. Thus, activity levels obtained represent the sum of endogenous and expressed FIX Padua. Especially at low expression levels, this could confound the ability to interpret data.

**Figure 6:**
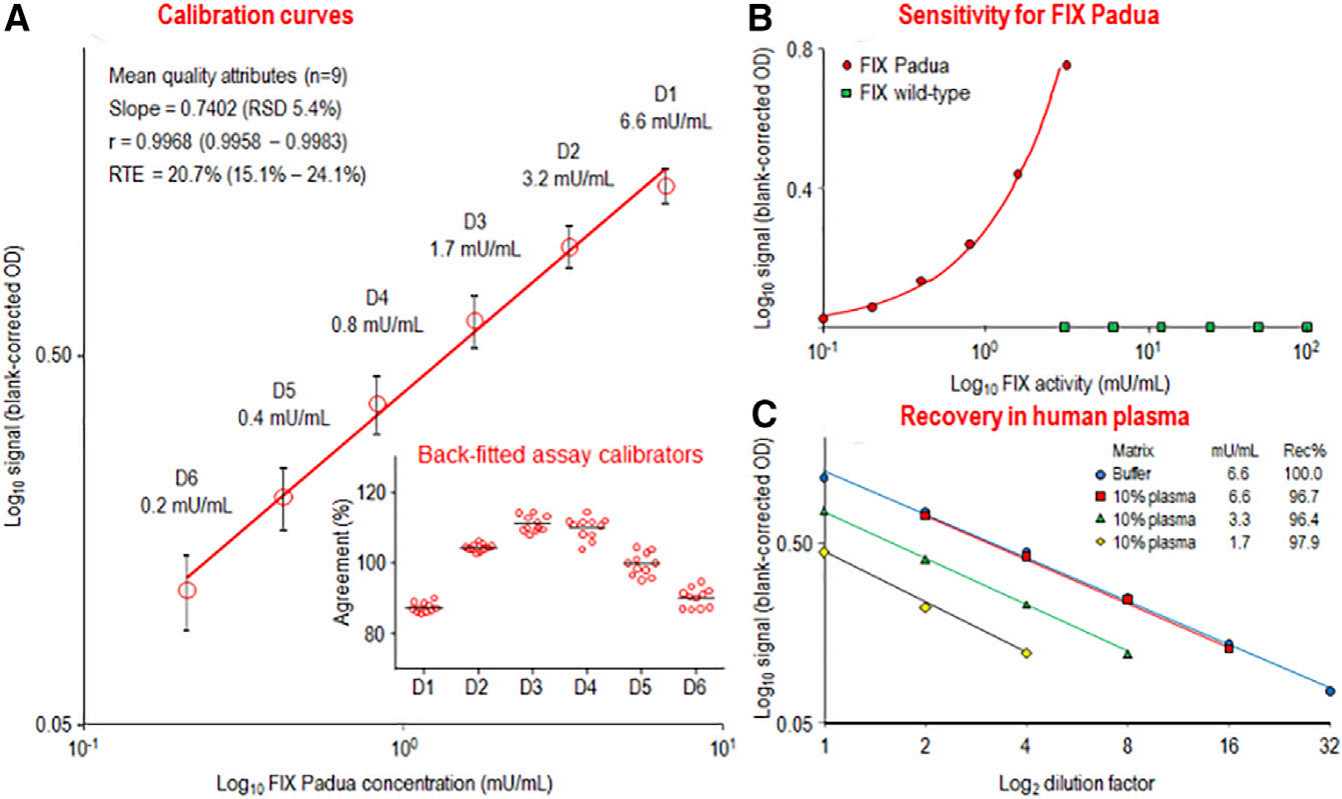
FIX Padua-Specific Chromogenic Assay (A) The mean (n = 9) calibration curve of the chromogenic FIX Padua activity assay. Error bars indicate the single SD of the blank-corrected ODs. (B) The assay’s sensitivity for FIX Padua is illustrated. (C) Comparison of the concentration-response curves for FIX Padua spiked to buffer and normal human plasma.

The mean assay calibration curve, shown in Figure 6A, confirmed that the basic concept of combining the FIX Padua-specific capture with the activity assay was feasible. The 6-point curve, prepared with an rFIX Padua preparation, ranged from 0.2 to 6.6 mU/mL. Thus, this activity assay was about 50 times more sensitive than that described for the measurement of FIX in solution.^23^ A typical FIX coagulation assay calibration curve ranges from 5 to 200 mU/mL in normal human plasma. Thus, compared with the calibration curve used for the FIX Padua chromogenic assay (0.2 to 6.6 mU/mL), our developed assay is also 25 times more sensitive than a clotting-based assay. The quality of curve fitting was adequate, meeting reproducibly the criteria of the EMA guideline for calibration curves of ligand-binding assays.^21^ Furthermore, the chromogenic assay proved to be highly specific for FIX Padua (Figure 6B): a 500-fold excess of human FIX over rFIX Padua did not elicit any signal. Moreover, citrated human normal plasma, diluted 1/10, was spiked to reach final rFIX Padua concentrations of 6.6, 3.3, and 1.7 mU/mL. Figure 6C shows the dilution-response curves, which were parallel to the assay calibration curve, which is prepared in buffer only. Recoveries ranged from 96.4% to 97.9%, clearly confirming the suitability of the assay. Similarly, Table 3 summarizes the data obtained for the three mouse plasma types investigated: two of them, CL57BL6 and nonobese diabetic/severe combined immunodeficiency (NOD/SCID) mice, contain normal levels of hemostatically functional mouse FIX. rFIX Padua was spiked at two levels, 0.33 and 3.31 mU/mL. The spiked citrated plasma samples were measured six times. rFIX Padua recoveries were within a 100 ± 7% range, thus clearly meeting the accuracy requirements of the EMA guideline for bioanalytical method validation.^21^ Similarly, intra-run precision, expressed as the RSD of the mean of six measurements, did not exceed 9%, even at the low rFIX Padua concentration of 0.33 mU/mL. Inter-run precision, determined by repeated measurements (n = 11) of an rFIX Padua sample, was 7.0%. These data confirmed the adequate performance of our approach for the selective activity measurement of FIX Padua.

**Table 3:** Recovery of rFIX Padua in Citrated Mouse Plasma Samples Measured with the FIX Padua Chromogenic Activity Assay

Mouse Type	Low Spike (0.33 mU/mL)		High Spike (3.3 mU/mL)	
	% Recovery	% Slope	% Recovery	% Slope
CL57BL6	102.0 ± 6.1	105.4	105.5 ± 3.6	107.8
NOD/SCID	100.5 ± 9.0	107.1	106.2 ± 4.8	105.6
FIX KO	93.9 ± 5.8	108.7	102.3 ± 3.6	106.1

Citrated mouse plasma samples were diluted 1/5 and spiked 1 + 1 with rFIX Padua to obtain the target concentrations. The recovery data represent the mean ± SD of six measurements on one plate; the relative slopes of the dilution-response curves, shown as a percentage of that of the calibration curve in the column % Slope, are the means of three measurements on three different plates.

## Discussion

The description of FIX Padua (R338L), a naturally occurring FIX gain-of-function mutation, laid the basis for the experimental drug BAX 335.^13^ This AAV8-based gene therapy product was designed to express this hyper-functional FIX in hepatocytes of hemophilia B patients following systemic administration. Conceptually, the higher specific activity of FIX Padua could provide efficacy, despite low FIX protein expression levels. In the context of gene therapy, a safety advantage may be conferred by the ability to express higher circulating FIX activity while exposing subjects to lower doses of the gene expression vector.

Such a treatment can be monitored by the measurement of FIX activity. This activity measurement is usually accompanied by the measurement of FIX protein levels using a FIX ELISA. Low FIX protein levels and the fact that some hemophilia B patients express FIX CRM^+^, an inactive FIX molecule, albeit measurable by ELISA, made it highly unlikely that analysis with a conventional ELISA could essentially contribute to a more profound understanding of the clinical data.

To overcome this limitation, an antibody selectively binding to FIX Padua was developed using phage display technology. The screening strategy, using a structural peptide and a linear peptide with the FIX Padua sequence, led to the identification of two binders with the former peptide and one binder (Ab42) with the latter peptide. However, the affinity of Ab42 to FIX Padua was at least one order of magnitude higher than those selected with the larger peptide and similar to that of a polyclonal anti-FIX antibody, and it did not show binding to human FIX, FII, or FX. Furthermore, Ab42 did not bind to FIX or any other protein in 20% human plasma, which was another prerequisite for the antibody to be used as capturing reagent for the analysis of plasma samples. The Ab42-derived Fab2 mini-antibody, therefore, proved well suited for the development of a FIX Padua-specific ELISA and a FIX Padua-specific chromogenic activity assay.

The FIX Padua-specific assays required the selective capture of FIX Padua from the complex matrix of citrated plasma. Therefore, considerable efforts were made to define a robust coating procedure for the Fab2 mini-antibody. The alkaline CB (pH 9.5) usually applied for the coating of whole IgG antibodies to the polystyrene surface was also advantageous for the Fab2 mini-antibody. The coating concentration selected (0.9 μg/mL) did not provide the highest response but resulted in the largest range of adequate signal to concentration relation. This was a decisive finding, because assay calibration curves should consist of a minimum of six non-zero concentrations following the EMA requirement on bioanalytical method validation.^21^ Notably, the finally defined robust coating procedure was applicable for ELISA and the activity assay.

Next to the coating procedure, the blocking of the wells is at least as important, especially when working with complex sample matrices like plasma.^24^ Optimally, the buffer used for this blocking step can be used also as sample DB, preventing adsorptive protein losses in dilute solutions, obtained when preparing the samples’ dilution series. BSA is known to have adequate blocking properties and was, therefore, used as an inert protein at a concentration of 5 mg/mL. This was effective for the ELISA and the activity assay, as demonstrated by the low signals obtained for the neat citrated plasma samples measured in the minimum dilution of 1/10 (data not shown).

The final HEPES-based sample DB also contained 10 mM Ca^2+^, as this cation turned out to have an important influence on the sensitivity of the ELISA. Like other vitamin-K-dependent coagulation proteins, FIX contains multiple Ca^2+^ binding sites—including the Gla domain, which binds approximately 10 Ca^2+^ molecules—and further binding sites in the first epidermal growth factor (EGF) domain and the serine protease domain.^25^ Ca^2+^ binding is known to result in profound structural changes of the molecule, which can explain the increased sensitivity observed in the presence of calcium.^26^ In addition, it cannot be excluded that Ca^2+^ also affected the conformation of the anti-FIX Padua Fab2 mini-antibody, considering the described Ca^2+^-dependent interaction of a monoclonal antibody with the Gla-domain-containing protein C.^27^ The selectivity of the rFIX Padua ELISA was repeatedly confirmed by the analysis of human normal plasma samples. No measurable response was detected, even though the plasma samples were measured at low dilutions. The suitability of the ELISA for the measurement of FIX Padua in citrated plasma samples was confirmed by spike-recovery studies, carried out in human normal and FIX-deficient plasma samples and in cynomolgus monkey plasma samples. None of these matrices showed substantial interference in the FIX Padua ELISA at the minimum dilution of 1/10. The recoveries of rFIX Padua were within a 100 ± 17% range, meeting the requirement of the EMA guideline on bioanalytical assay validation.^21^ Furthermore, spike recovery carried out for eight clinical plasma samples from the BAX 335 trial at the minimum dilution of 1/10 showed recoveries ranging from 86.7% to 100.7%. In addition, the dilution-response curves of the plasma samples showed adequate parallelism to those of the corresponding calibration curves. Finally, applying the assay to a representative set of clinical samples from the BAX 335 trial revealed a correlation between the deduced FIX Padua activity levels and measured FIX clotting activity, whereas no correlation was seen for the FIX wild-type and FIX Padua ELISAs. These results, thus, proved the suitability of the FIX Padua ELISA, described here, for the analysis of clinical samples from gene therapy trials, as it contributed to unambiguously identify FIX Padua expression, even at low expression levels.

It is worth noting that, among the other three ongoing hemophilia B gene therapy trials, one study uses FIX Padua already, and another is anticipated to switch from wild-type FIX to the Padua variant.^28^, ^29^ Thus, it appears that the hyperactive FIX Padua has become the gold standard in hemophilia B gene therapy;^30^ our developed FIX Padua ELISA would provide an important analytical tool to support those clinical developments, as it allows a precise quantification of transgene expression regardless of the subjects’ genetic background.

The availability of the anti-FIX Padua Fab2 mini-antibody made it possible to also develop a chromogenic activity assay. Captured FIX Padua could be measured with the slightly adapted commercially available chromogenic assay. In this assay, FIX is activated by FXIa to yield FIXa, which, in the presence of FVIIIa and phospholipid, is able to efficiently activate FX. The resulting activated FX is measured with a chromogenic substrate. Surprisingly, the Fab2 mini-antibody-captured rFIX Padua showed good activity in this test system. Since activation and complex formation of FIX Padua was apparently not hampered considerably, there was no steric hindrance imposed by the binding of the antibody to an epitope of FIX Padua that included amino acid L338. Our approach of combining antibody capture with selective activity measurement of the mutant protein variant resembles that described for the measurement of PEGylated factor VIII,^31^ using the polyethylene glycol (PEG) moiety for the selective capture of the modified protein. The sensitivity of the assay calibration curve (0.2 mU/mL) of the chromogenic FIX assay was 50 times higher than that described.^23^ Spike-recovery studies in citrated normal human plasma and three types of mouse plasma samples demonstrated adequate recoveries with 100 ± 10%. Precision analyses with spiked citrated mouse plasma samples provided RSDs of no higher than 9%, which is in the same magnitude as that published for the use of the less complex chromogenic assay in human plasma.

All in all, the data confirmed the successful development of a FIX Padua-specific ELISA that can be applied for the measurement of this hyper-functional FIX protein in human and cynomolgus monkey plasma samples. Furthermore, the Fab2 mini-antibody was also successfully used to set up a selective chromogenic activity assay and was shown to perform adequately in human and mouse plasma samples. These data highlight the progress in the generation of tailored biological antibody-based reagents, as it was possible to develop an antibody discriminating the single amino acid exchange variant FIX Padua from normal FIX protein. We anticipate that the approach described here could be of relevance also for other gene therapies that are aimed at tackling hard-to-treat indications where expression of highly active enzymes would be required to achieve therapeutically relevant levels of the transgene product.

## Materials and Methods

### Biological Materials

Sheep anti-human FIX IgG (A-COA F9-1030A) and streptavidin peroxidase (P0397) were from CoaChrom Diagnostica (Vienna, Austria) and DakoCytomation (Vienna, Austria), respectively. Anti-FIX IgG was biotinylated with sulfo-NHS-LC-biotin (Fisher Scientific Austria, Vienna, Austria) according to the manufacturer’s instructions, resulting in a molar biotinylation degree of 2.8. Anti-FIX Padua Fab2 mini-antibody #AbD24742.1 (Ab42) was generated using the technology provided by Bio-Rad AbD Serotec (Vienna, Austria). rFIX Padua, expressed in CHO cells, was purified to a specific clotting activity of 2,310 IU/mg of protein. The chromogenic FIX activity test kit BIOPHEN FIX was from Hyphen BioMed (CoaChrom Diagnostica, Vienna, Austria). Pooled human reference plasma (Precision Biologics, CCNRP-05; CoaChrom Diagnostica, Vienna, Austria) and rFIX RIXUBIS (Baxalta, Vienna, Austria) were used as FIX wild-type sources; FIX-deficient plasma was from Hyphen BioMed (CoaChrom Diagnostica, Vienna, Austria).^32^ Citrated cynomolgus monkey plasma samples were obtained from Covance Laboratories (Münster, Germany); citrated mouse plasma samples (C57BL6, NOD/SCID, and FIX knockout [KO] mice) were obtained from the Baxalta in-house animal facility (Vienna, Austria). Citrated plasma samples from the phase 1/2 open-label clinical trial BAX 335 (ClinicalTrials.gov: NCT01687608) were used to investigate the feasibility of the FIX Padua ELISA; informed consent to conduct these studies was obtained from all patients. Further chemicals and reagents are described with the respective methods.

### Generation and Selection of the Anti-FIX Padua Fab2 Mini-antibody

Phage display technology with a HuCAL PLATINUM library and CysDisplay technology was used for generation of the FIX Padua-specific antibody fragment. In particular, Fab2 mini-antibodies consisting of bivalent Fab mini-antibodies conjugated with an alkaline phosphatase domain for dimerization were expressed. Screening for FIX Padua binders was carried out with three different strategies. Two used FIX Padua-specific peptides: the first represented the linear amino acid sequence DRATCL*L*STKFT, and the second (LVDRATCL*L*STKFTIYNNMFCAGFH) contained a disulfide bridge. Another strategy was based on using a combination of the peptides and a recombinant full-length FIX Padua. Wild-type FIX binders were identified with the corresponding peptides of FIX wild-type. The biotinylated peptides or rFIX Padua were coupled to Dynabeads M-280 Streptavidin (“bead-based panning”) or passively adsorbed to plates (“solid-phase panning”). After three panning rounds, the enriched pools of Fab genes were isolated and cloned into expression vectors to generate Fab mini-antibodies. Selected anti-FIX Padua binders were tested with ELISA to confirm specific FIX Padua binding in buffer, in the presence of normal plasma levels of purified wild-type FIX (5 μg/mL), and in the presence of 20% citrated human plasma. Direct ELISAs with purified coagulation factors II and X were used to investigate the cross-reactivity of candidates against these closely related proteins. Affinities of the lead molecule Ab42 to FIX Padua and of the sheep polyclonal anti-FIX antibody (SAFIX-AP-150, CoaChrom Diagnostica) to FIX was determined by surface plasmon resonance technology on Biacore T200 equipment (GE Healthcare, Vienna, Austria).

### Development of Coating Conditions

Relevant ELISA parameters were investigated, including coating conditions and the composition of the DB.^33^ Emphasis was placed on coating concentration and pH, since a Fab2 mini-antibody was used instead of full-length IgG, which misses the Fc portion of IgG.

Coating concentration was addressed by coating Ab42 solutions of 0.47, 0.94, 1.9, and 4.7 μg/mL PBS at +4°C overnight. A serial dilution series of rFIX Padua (1.45 to 116 ng/mL), diluted in PBS with 0.05% Tween 20 (PBST) containing 0.5% biotin-free BSA, 2 mM benzamidine, and 10 mM EDTA was analyzed. All other conditions were those of the final method, except Ca^2+^. The optimal pH was determined by comparing the efficiency of Fab2 mini-antibody (1.9 μg/mL) coating in a neutral buffer (PBS; pH 7.2) and an alkaline buffer (carbonate; pH 9.5). All other conditions were those of the final method, except Ca^2+^.

### Description of the FIX Padua ELISA

Nunc Maxisorp F96 flat-bottom 96-well plates (VWR, Vienna, Austria) were used. Buffer salts (all analytical grade) were from VWR (Vienna, Austria); Tween 20 (enzyme immunoassay [EIA] grade) was from Bio-Rad (Vienna, Austria); biotin-free BSA was from Carl Roth (Karlsruhe, Germany); the peroxidase substrate SureBlue was from KPL (Medac, Hamburg, Germany); HEPES, CaCl_2_ × 2 H_2_O, and benzamidine hydrochloride were from Sigma (Vienna, Austria); and 3 N sulfuric acid was from Alfa Aesar (now part of Thermo Fisher Scientific, Karlsruhe, Germany). The following buffers were used: CB, 0.1 M carbonate (pH 9.5); PBS buffer (pH 7.2); washing buffer, PBST; and DB, 100 mM HEPES, 100 mM NaCl, (pH 7.2), 10 mM CaCl_2_, 10 mM benzamidine, 0.05% Tween 20, and 0.5% biotin-free BSA. Plates were coated with 100 μL Ab42 per well, diluted 1/500 with CB (0.9 μg/mL), at +4°C overnight; coating was terminated by washing with PBST. Wells were blocked with 200 μL DB per well at room temperature (RT) for 60 min. After a washing step, all wells were filled with 100 μL DB, before serial dilution series were prepared in duplicates directly on the plate and incubated at RT for 60 min. After a washing step, sequential additions of biotinylated detection antibody (1/500 in DB, at RT, for 60 min) and streptavidin peroxidase (1/4,000 in DB, at RT, for 30 min) completed the incubation steps, with each step terminated by a washing step. Bound peroxidase activity was determined with SureBlue and 3 N sulfuric acid and by measuring the plate at 450 nm. The six-point calibration curve (range = 0.85 to 27.1 ng/mL) was constructed on each plate with purified rFIX Padua applying a log-log fitting.

### Description of the Chromogenic FIX Padua Activity Assay

The chromogenic FIX test is described for FIX activity measurement in solution. Here, it was modified for the measurement of antibody-adsorbed FIX Padua. Ab42-coated and blocked plates and dilution series were prepared as described earlier. The six-point calibration curve ranged from 0.2 to 6.6 mU/mL. Basically, the assay was carried out according to the manufacturer’s instructions,^23^, ^34^ with the following modifications: altered reagent volumes (40 μL instead of 50 μL), altered incubation temperature (RT instead of +37°C), and prolonged incubation times to compensate for the difference in temperature. Briefly, 40 μL per well of Tris-BSA reaction buffer (R4) was added to the washed wells, followed by 40 μL reagent R1 (human FX and FVIII) per well. The plate was incubated for 10 min before 40 μL activation reagent R2 (human factor XIa, human thrombin and synthetic phospholipids) was added per well and incubated for 15 min (optimized in an incubation times study). Finally, 40 μL reagent R3 (chromogenic substrate) was added per well and incubated for 15 min. Then, the plate was measured at 405 nm. The calibration curve was obtained by log-log fitting of the blank-corrected ODs and the FIX Padua concentrations of the assay calibrators. Assay performance was checked in citrated human normal plasma and three types of citrated mouse plasma (C57BL6, NOD/SCID, and FIX KO mice).

## Author Contributions

Conceptualization, D.V. and H.R.; Methodology, A.W., A.E., D.V., and R.P.; Investigation, A.W. and A.E.; Writing – Original Draft, A.W.; Writing – Review & Editing, A.W., A.E., D.V., R.P., P.E.M., F.S., and H.R; Visualization, A.W.

## Conflicts of Interest

This work was funded by Shire. A.W., A.E., D.V., R.P., F.S., and H.R are full-time employees of Shire. P.E.M. completed the work during his employment with Shire and is no longer at Shire.

## References

[1] Mannucci P.M., Tuddenham E.G. (2001). The hemophilias--from royal genes to gene therapy. N. Engl. J. Med..

[2] Powell J.S. (2015). Longer-acting clotting factor concentrates for hemophilia. J. Thromb. Haemost..

[3] Taylor J.A., Kruse-Jarres R. (2016). A new era for hemophilia B treatment. Blood.

[4] Powell J.S., Pasi K.J., Ragni M.V., Ozelo M.C., Valentino L.A., Mahlangu J.N., Josephson N.C., Perry D., Manco-Johnson M.J., Apte S., B-LONG Investigators (2013). Phase 3 study of recombinant factor IX Fc fusion protein in hemophilia B. N. Engl. J. Med..

[5] Collins P.W., Young G., Knobe K., Karim F.A., Angchaisuksiri P., Banner C., Gürsel T., Mahlangu J., Matsushita T., Mauser-Bunschoten E.P., paradigm 2 Investigators (2014). Recombinant long-acting glycoPEGylated factor IX in hemophilia B: a multinational randomized phase 3 trial. Blood.

[6] Santagostino E., Martinowitz U., Lissitchkov T., Pan-Petesch B., Hanabusa H., Oldenburg J., Boggio L., Negrier C., Pabinger I., von Depka Prondzinski M., PROLONG-9FP Investigators Study Group (2016). Long-acting recombinant coagulation factor IX albumin fusion protein (rIX-FP) in hemophilia B: results of a phase 3 trial. Blood.

[7] Kay M.A., Rothenberg S., Landen C.N., Bellinger D.A., Leland F., Toman C., Finegold M., Thompson A.R., Read M.S., Brinkhous K.M. (1993). In vivo gene therapy of hemophilia B: sustained partial correction in factor IX-deficient dogs. Science.

[8] Lozier J.N., Brinkhous K.M. (1994). Gene therapy and the hemophilias. JAMA.

[9] Benveniste O. (2012). Gene therapy, an ongoing revolution. Blood.

[10] Connelly S., Kaleko M. (1997). Gene therapy for hemophilia A. Thromb. Haemost..

[11] Tuddenham E. (2012). Gene therapy for haemophilia B. Haemophilia.

[12] Nathwani A.C., Tuddenham E.G.D., Rangarajan S., Rosales C., McIntosh J., Linch D.C., Chowdary P., Riddell A., Pie A.J., Harrington C. (2011). Adenovirus-associated virus vector-mediated gene transfer in hemophilia B. N. Engl. J. Med..

[13] Simioni P., Tormene D., Tognin G., Gavasso S., Bulato C., Iacobelli N.P., Finn J.D., Spiezia L., Radu C., Arruda V.R. (2009). X-linked thrombophilia with a mutant factor IX (factor IX Padua). N. Engl. J. Med..

[14] Monahan P.E., Sun J., Gui T., Hu G., Hannah W.B., Wichlan D.G. (2015). Employing a gain-of-function factor IX variant R338L to advance the efficacy and safety of hemophilia B human gene therapy: preclinical evaluation supporting an ongoing adeno-associated virus clinical trial. Hum. Gene Ther..

[15] Cantore A., Nair N., Della Valle P., Di Matteo M., Màtrai J., Sanvito F., Brombin C., Di Serio C., D’Angelo A., Chuah M. (2012). Hyperfunctional coagulation factor IX improves the efficacy of gene therapy in hemophilic mice. Blood.

[16] Finn J.D., Nichols T.C., Svoronos N., Merricks E.P., Bellenger D.A., Zhou S., Simioni P., High K.A., Arruda V.R. (2012). The efficacy and the risk of immunogenicity of FIX Padua (R338L) in hemophilia B dogs treated by AAV muscle gene therapy. Blood.

[17] Monahan P.E. (2015). Gene therapy in an era of emerging treatment options for hemophilia B. J. Thromb. Haemost..

[18] High K.A., Anguela X.M. (2016). Adeno-associated viral vectors for the treatment of hemophilia. Hum. Mol. Genet..

[19] Spencer H.T., Riley B.E., Doering C.B. (2016). State of the art: gene therapy of haemophilia. Haemophilia.

[20] Bertina R.M., Veltkamp J.J. (1978). The abnormal factor IX of hemophilia B+ variants. Thromb. Haemost..

[21] European Medicines Agency. (2011). Guideline on bioanalytical method validation. (EMEA/CHMP/EWP/192217/2009). http://www.ema.europa.eu/docs/en_GB/document_library/Scientific_guideline/2011/08/WC500109686.pdf.

[22] Furie B., Furie B.C. (1988). The molecular basis of blood coagulation. Cell.

[23] Peyrafitte M., Vissac A.M., Amiral J. (2009). New standardized chromogenic assays for the automated measurements of FIX or FIXa in plasma and therapeutic concentrates. https://www.coachrom.com/fileadmin/user_upload/docs/publikationen/FIX_PostAbs.pdf.

[24] Kemeny D.M. (1991). A practical guide to ELISA.

[25] Persson K.E.M., Astermark J., Björk I., Stenflo J. (1998). Calcium binding to the first EGF-like module of human factor IX in a recombinant fragment containing residues 1-85. Mutations V46E and Q50E each manifest a negligible increase in calcium affinity. FEBS Lett..

[26] Hansson K., Stenflo J. (2005). Post-translational modifications in proteins involved in blood coagulation. J. Thromb. Haemost..

[27] Stearns D.J., Kurosawa S., Sims P.J., Esmon N.L., Esmon C.T. (1988). The interaction of a Ca2+-dependent monoclonal antibody with the protein C activation peptide region. Evidence for obligatory Ca2+ binding to both antigen and antibody. J. Biol. Chem..

[28] George L.A., Sullivan S.K., Giermasz A., Rasko J.E.J., Samelson-Jones B.J., Ducore J., Cuker A., Sullivan L.M., Majumdar S., Teitel J. (2017). Hemophilia B gene therapy with a high-specific-activity factor IX variant. N. Engl. J. Med..

[29] Miesbach W., Meijer K., Coppens M., Kampmann P., Klamroth R., Schutgens R., Tangelder M., Castaman G., Schwäble J., Bonig H. (2018). Gene therapy with adeno-associated virus vector 5-human factor IX in adults with hemophilia B. Blood.

[30] Lillicrap D. (2017). FIX it in one go: enhanced factor IX gene therapy for hemophilia B. Cell.

[31] Weber A., Engelmaier A., Mohr G., Haindl S., Schwarz H.P., Turecek P.L. (2017). Selective functional activity measurement of a PEGylated protein with a modification-dependent activity assay. J. Pharm. Biomed. Anal..

[32] Valentino L.A. (2014). The role of Rixubis™ in the treatment of hemophilia B. Immunotherapy.

[33] Jordan B. (2000). How to set up an ELISA. Methods Mol. Med..

[34] Wagenvoord R., Hendrix H., Tran T., Hemker H.C. (1990). Development of a sensitive and rapid chromogenic factor IX assay for clinical use. Haemostasis.

